# Reconstruction of metastatic acetabular defects using a modified Harrington procedure: Good outcome after surgical treatment of 70 patients

**DOI:** 10.3109/17453674.2015.1077308

**Published:** 2015-11

**Authors:** Panagiotis Tsagozis, Rikard Wedin, Otte Brosjö, Henrik Bauer

**Affiliations:** Section of Orthopaedics, Department of Molecular Medicine and Surgery, Karolinska Institute and Department of Orthopaedics, Karolinska University Hospital, Stockholm, Sweden

## Abstract

**Background and purpose —** Metastases engaging the acetabulum result in significant disability. We investigated the outcome after curettage and reconstruction of the defect with a protrusio cage, retrograde screws, and a cemented total hip arthroplasty.

**Patients and methods —** We retrospectively identified 70 consecutive patients who were surgically treated for metastatic disease of the acetabulum between 1995 and 2012 using the above technique. The type of primary tumor, extent of the disease, degree of acetabular erosion, and type of implant used were identified. Patient and implant survival, complications, and functional outcome were recorded.

**Results —** There were no mortalities in the perioperative period (30 days after surgery). Median overall patient survival was 12 months. Prosthesis survival was 92% at 1 year and 89% at 5 years. One third of the patients suffered a complication, the most frequent one being dislocation. The functional outcome was good. Multiple skeletal or visceral metastases and specific types of cancer were associated with poor patient survival.

**Interpretation —** Reconstruction of metastatic acetabular defects using a protrusio cage stabilized with retrograde screws and a cemented total hip arthroplasty is a safe procedure that provides efficient relief of symptoms. Patients with extensive disease, especially when diagnosed with specific types of cancer, have a very poor prognosis. The complication rate is substantial, the most frequent being dislocation. However, revision surgery is seldom required and prosthesis survival is high.

Metastatic involvement of the pelvis is common, second only to spinal involvement (Clain 1965, Picci 2014). Engagement of the acetabular region entails a major risk for pathological fracture due to the high mechanical loads, and it is often accompanied by considerable pain and restriction in ambulation. Nonoperative treatments such as radiotherapy, chemotherapy, hormonal therapy, and bisphosphonates are as a rule insufficient to reduce pain and restore ambulation when there is mechanical instability (Levy et al. 1982, Wedin 2001). Surgery entails the removal of pathological tissue and restoration of the iliofemoral weight-bearing axis by reconstructing the remaining skeletal defect. Harrington (1981) classified metastatic disease of the acetabulum and described the surgical technique of reconstructing the pelvic ring with multiple pins, cement, cage, and a cemented total hip replacement. Since then, the method has been validated and modifications of the technique have been proposed (Kunisada and Choong 2000, Marco et al. 2000, Nilsson et al. 2000, Vielgut et al. 2013, Shahid et al. 2014). Some authos have proposed a simpler surgical technique, namely the use of retrograde screws placed through the protrusio cage to transmit weight loads to structurally intact bone, which follows the principles outlined by Harrington (Stark and Bauer 1996, Vena et al. 1999, Clayer 2010, Hoell et al. 2012). However, there are still too few data, as all series have involved a small number of patients. Nevertheless, the results suggest that surgery can provide pain relief and improvement in the ambulatory capacity of the patient, although it is accompanied by a considerable risk of complications.

At our institution, metastatic lesions in the periacetabular region are usually treated with curettage of the tumor and reconstruction with a protrusio cage and total hip replacement. We use screws placed through the cage in retrograde fashion, and do not place Steinman pins. We have found this method to be sufficient for most lesions, and we use “ice-coned” implants only when there is insufficient bone stock to attach the cage, or if there is pelvic discontinuity. In this study, we present our experience in using the above technique in 70 consecutive patients who were operated between 1995 and 2012.

## Patients and methods

The pathological fracture database of our department consists of prospectively collected data. We retrospectively reviewed the database and retrieved data from 70 consecutive patients (36 of them men; 70 acetabulae) who were operated with curettage and reconstruction with a protrusio cup and cemented total hip replacement. Mean follow-up was 21 months, and 68 patients had died at last follow-up. Median age at operation was 64 (40–86) years. Prostate cancer was the primary tumor in 24 patients, breast cancer in 20, myeloma in 6, lung cancer in 5, genito-urinary cancer in 4, kidney carcinoma in 4, and other/unknown primary cancer at diagnosis in 3 patients. There were 22 Harrington class-2 defects and 40 class-3 defects, whereas a reliable classification could not be performed in 8 patients. A pathological fracture was apparent in 62 patients. 19 patients were diagnosed with a single skeletal metastasis, 26 had numerous skeletal lesions, and 25 had generalized disease (visceral involvement). Radiotherapy had been given to 41 patients prior to operation, and to 11 patients postoperatively. The decision to give adjuvant radiotherapy was individualized, and the presence of limited sclerotic metastases or poor expected survival was generally considered to be a contraindication for radiotherapy.

Plain radiographs, computed tomography scans, or magnetic resonance tomography was used for diagnosis and follow-up. The Müller cage was used in 69 patients and the Burch-Schneider cage was used in 1 patient. Regarding the femoral stems used for reconstruction, the Spectron (Smith and Nephew) was used in 32 patients, the MS-30 stem (Zimmer) was used in 19, the Charnley (De Puy) was used in 7, and the Lubinus SPII (Link, Germany) was used in 12. A 22-mm femoral head was used in 7 patients, a 28-mm head in 42 patients, a 32-mm head in 20 patients, and a 50-mm constrained one in 1 patient. The posterolateral approach to the hip joint and acetabulum was used in 66 patients and the anterolateral approach was used in 4. Preoperative embolization was used in only 1 patient.

**Figure 1.**
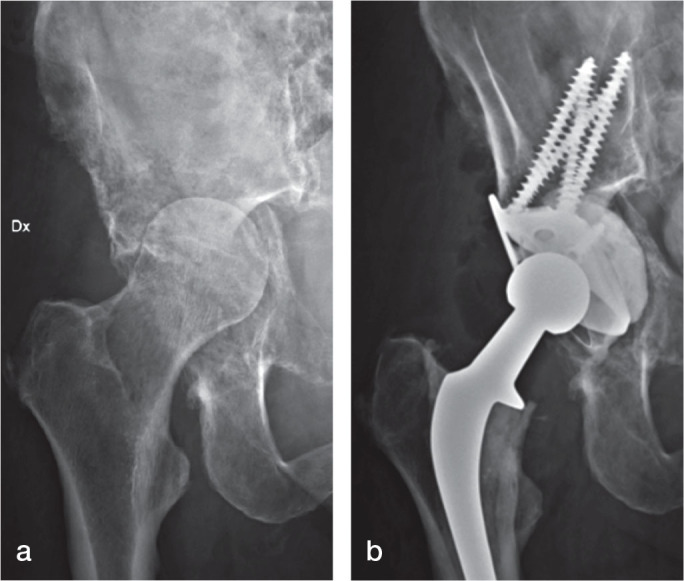
A 66-year-old woman with metastatic destruction of the left acetabulum from breast cancer, and protrusion of the caput (a). Reconstruction was done with a Müller cage, multiple retrograde screws, and a cemented total hip arthroplasty using a Spectron femoral stem (b).

The metastasis was curetted and the protrusio cage was adapted with multiple screws in order to gain purchase to pelvic bone of adequate quality. The resulting defect after curettage was filled with polymethylmethacrylate and a polyethylene cup was cemented. The femoral stem was then cemented using conventional technique (Figure 1). Routine antibiotic prophylaxis was a 3-dose regimen of flucloxacillin or clindamycin. Subcutaneous low-molecular-weight heparin was given for 4 weeks. The postoperative scheme included full weight bearing. The outcome of surgery regarding pain and ambulatory capacity of the patients was evaluated approximately 2 months after the operation, with additional follow-up scheduled if necessary. Pain was graded on a 5-degree scale (0: none; 1: mild; 2: intermittent moderate; 3: constant moderate; and 4: severe) and ambulation was graded on a 4-degree scale: (0: bedridden; 1: confined to a wheelchair; 2: partial weight bearing with walking aids; and 3: ambulating without walking aids). Complications were either classified as prosthetic (dislocation or failure of the implant) or biological (symptomatic local tumor progression and loosening, local deep infection, wound dehiscence, or any systemic side effects of surgery). Revision of the prosthesis was defined when any of the cemented parts of the prosthesis or the protrusio cage were either revised or removed.

Survival analysis was done according to the Kaplan-Meier technique, and comparisons were made using the log-rank test. Comparisons between groups were done using Pearson’s chi-square test. All tests were double-sided, and any p-value of ≤ 0.05 was considered significant. 95% confidence intervals (CIs) were calculated for survival of patient groups. Statistical analysis of the data was carried out using SPSS version 18 and MedCalc software (MedCalc, Ostend, Belgium). The study conformed to institutional review board requirements.

## Results

**Figure 2.**
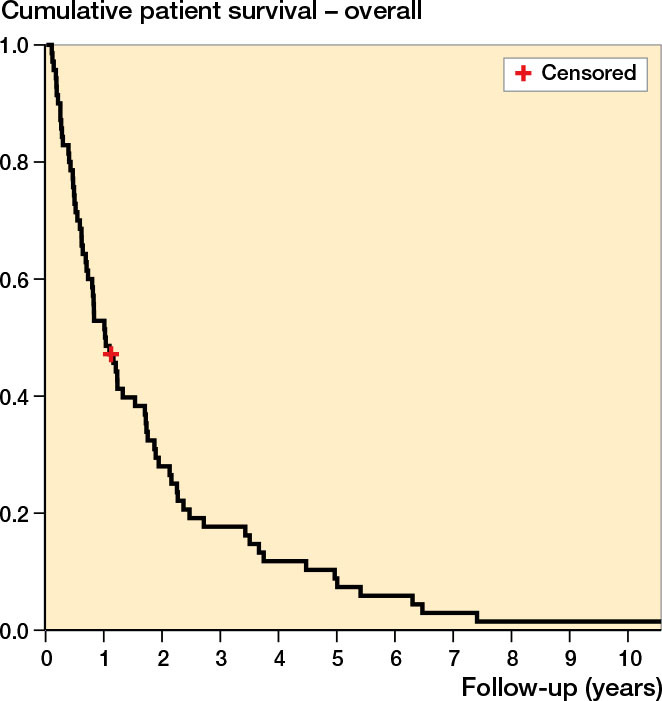
Overall survival of 70 patients surgically treated for acetabular metastasis with a protrusion cage and total hip arthroplasty. Median overall survival of the cohort was 12 months.

**Figure 3.**
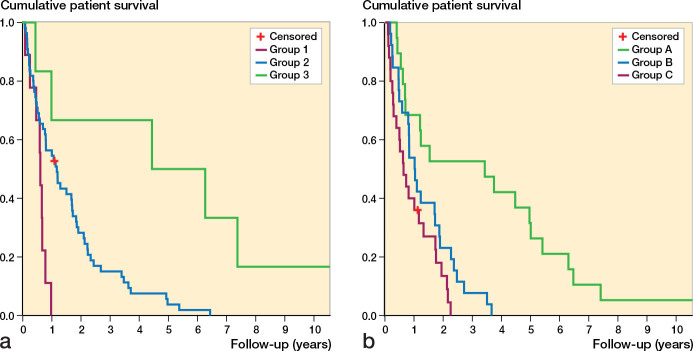
Overall survival in the 70 patients based on the type of the primary tumor (a) and the extent of the metastatic disease (b). Certain primary tumors and extensive metastatic involvement (multiple skeletal or visceral metastases) give a poor prognosis. Statistically significant differences between the groups were seen in both cases (p ≤ 0.05). For groups, see text.

Median operative time was 95 (59–178) min, and the median number of screws used to stabilize the protrusio cup was 5 (2–7). Median follow-up time was 12 (1–205) months. Median survival of the cohort was 12 (95% CI: 7–16) months and mean survival was 21 (CI: 15–29). Survivorship at 1 year after reconstruction was 49% (CI: 38–60), with survivorship at 5 years being only 7% (CI: 2–17) (Figure 2). Survival was dependent on 2 main factors: the type of the primary tumor and the extent of the disease. Patients diagnosed with melanoma, genito-urinary cancer, thyroid cancer, or unknown primary tumor at diagnosis (group 1) had the worse prognosis, with a mean survival of 7 (CI: 6–8) months. Patients with prostate, breast, renal, lung, and head-and-neck cancer (group 2) had a mean survival of 19 (CI: 9–20) months, whereas patients with hematopoietic malignancies such as multiple myeloma and plasmocytoma (group 3) had a mean overall survival of 72 (CI: 12–87) months. A Kaplan-Meier analysis of the effect of tumor type on survival is presented in Figure 3a. Furthermore, patients presenting with localized disease had a significantly better prognosis (group A), with an estimated mean survival of 44 (CI: 22–59) months, than those with multiple skeletal involvement (group B) or generalized metastatic disease (group C), who had a mean survival of 15 (CI: 9–20) months and 10 (CI: 3–14) months, respectively (Figure 3b). Interestingly, the 30-day perioperative mortality of the series was 0%.

Overall prosthesis survival was 92% (CI: 87–99) at 1 year and 89% (CI: 81–97) at 5 years. Implant revision took place in 5 patients: 3 cups were revised, 1 because of symptomatic loosening and 2 because of recurrent dislocations. Implants were extracted in 2 patients; 1 had a deep infection and the other had painful recurrent dislocations. There were no statistically significant differences in prosthesis survival between Harrington class-2 defects and class-3 defects.

Surgery resulted in pain relief in all patients, and better ambulation in the majority of them. On a 5-degree scale, the average difference in pain scale was +2.7, whereas in terms of ambulation, the observed effects were also positive (+0.81) but not as profound. Furthermore, there were 3 patients who had negative results regarding ambulation: 2 of them after removal of the implant (resulting in excision arthroplasty of the hip), and 1 after repeated surgery for persistent deep infection.

There were 27 complications in 23 patients. Prosthetic complications were documented in 13 patients and biological complications in 10. Dislocation of the hip accounted for all the mechanical complications (n = 13). Repeated dislocations (≥ 3) occurred in 4 patients. There was no correlation between the use of prosthetic heads of large diameter (32 mm or more) rather than smaller ones (28 mm or less) and the risk of dislocation. Deep infection was documented in 7 patients. Symptomatic aseptic loosening and local tumor progression were observed in 3 patients, whereas minor asymptomatic findings of tumor progression and loosening were documented in 4 patients. Patients who survived for more than 1 year had a greater likelihood of presenting with prosthetic complications (as opposed to biological complications) than patients who survived for less than 12 months (p = 0.03) (Table).

**Table:** Type of complication and patient survival, dichotomized at 1 year. Patients who survive for more than 1 year are more likely to present with prosthetic complications rather than biological ones, whereas patients who survive for less than 1 year are more likely to suffer from biological complications (p = 0.03)

Survival < 1 year	Survival > 1 year	Total
Mechanical complications	5	8	13
Biological complications	9	1	10
Total	14	9	23

## Discussion

Engagement of the acetabulum and subsequent pathological fracture is a frequent cause of morbidity in cancer patients. Reconstruction of the defect in order to transmit loads to structurally intact parts of the pelvic ring is a challenge for the surgeon.

We have used a simplified Harrington technique, namely the insertion of retrograde screws through a protrusio cage combined with hip arthroplasty, to treat most of the patients who present with metastases and pathological fractures of the acetabulum (Stark and Bauer 1996). Here we report our medium-term results from the largest cohort of patients to be described to date who were treated for metastatic lesions of the acetabulum using a single method. One strength of the study was that factors that affected patient survival and complications were analyzed systematically. The main limitation was that the study was retrospective. Furthermore, it covered a period of 17 years (even though data in the database were collected prospectively), with a variety of implants being used. Also, we lacked a validated instrument to evaluate functional outcome.

Overall, we have found this technique to be simple and reliable. We resort to more complex solutions, such as “ice-coned” implants, only in the relatively few cases of extensive defects or pelvic discontinuity. Although one third of the patients had a symptomatic complication, the good functional results and the unexpectedly low perioperative mortality justify the view that surgery should be offered even to end-stage patients in order to provide pain relief and improved ambulation. Indeed, median survival after surgery was approximately 1 year, which is in line with previously published data (Tillman et al. 2008, Clayer 2010, Shahid et al. 2014). However, certain primary tumors and widespread metastatic disease were associated with a very poor prognosis, and this should be kept in mind when allocating patients with acetabular metastases to surgery or nonoperative treatment, especially when the clinical symptoms are moderate. We believe that our data on expected survival will be helpful in guiding the clinical decision.

Dislocation was the most frequent complication, and the main reason for revision surgery. Previous studies have also shown a high dislocation risk (Stark and Bauer 1996, Vena et al. 1999, Clayer 2010). The use of heads of larger diameter was not associated with a reduced risk of dislocation, which is mainly attributed to the extensive release in order to gain access to and curette the acetabular lesion, and also to the poor condition of the soft tissues after radiotherapy and cachexia associated with cancer. Moreover, the lack of anatomical landmarks as a result of bone erosion by the tumor is an obstacle to proper placement of the components, while orientation of the protrusio cup in the eroded acetabulum is often a compromise between the optimal expected position and the need to gain purchase in the remaining bone with as many screws as possible. Nowadays, the existence of constrained cups of large diameter is a promising solution, and we encourage their routine use in this scenario. Likewise, the anterolateral approach to the joint can probably reduce the risk of dislocation, and modern intraoperative navigation techniques may be helpful.

Other biological failures were also noted, with infection rates being around 10%. This can be explained by predisposing factors such as previous radiotherapy and cachexia (13). Notably, infection rarely resulted in revision surgery, as antibiotics and minor soft-tissue procedures are sufficient in this patient cohort where survival is short. Nonetheless, efforts should be focused on optimizing known factors that affect susceptibility to infection such as nutrition status, cessation of smoking, and blood glucose control in patients with diabetes, as well as following strict hygiene routines and using proper antibiotic prophylaxis (Merollini et al. 2013). Generally, the complication rate of one third is in accordance with previous findings (Wunder et al. 2003).

The surgical technique described is less invasive than the original Harrington-type reconstruction, which requires an additional iliac incision, which is something that should be taken into consideration in this frail population. One additional advantage of the method is that local side effects from erroneous placement of the pins or late complications from soft-tissue irritation are avoided. There is concern regarding local progression of the disease, which can lead to loosening of the construct, as the use of retrograde screws may be mechanically inferior to the use of multiple Steinman pins when there is local progression. According to our observations, this is generally asymptomatic and as a rule the method is sufficient for the treatment of the majority of acetabular defects, taking into account the short expected survival of the patients. This is corroborated by the observation that prosthesis survival is excellent, and does not differ significantly between extensive (Harrington class-3) defects and more restricted (Harrington class-2) involvement.

In summary, our data suggest that reconstruction of metastatic acetabular defects using a protrusio cage, retrograde screws, and a cemented total hip artroplasty is a safe procedure that results in good functional outcome. Overall survival of the patients is poor, and some of them have a very unfavorable prognosis, a fact that should be considered and individually discussed in decision making regarding surgery. Although the operation is associated with a considerable complication rate, as all surgery for pelvic metastases has been so far, the prosthesis revision rate is low. Current advances in implant design promise to reduce the risk of mechanical complications even further.
